# Cortical and neuromuscular features during a side-lying hip abduction drop task are associated with single-leg standing balance

**DOI:** 10.1016/j.ibneur.2026.06.019

**Published:** 2026-06-29

**Authors:** Kota Kitamura, Naoya Shimoda, Utaha Aoki, Kota Maeda, Hayato Wakabayashi, Junya Miyazaki, Takayuki Kodama, Hayato Shigetoh

**Affiliations:** Department of Physical Therapy, Faculty of Health Science, Kyoto Tachibana University, 34 Yamada-cho, Oyake, Yamashina-ku, Kyoto 607-8175, Japan

**Keywords:** Anticipatory postural adjustment, Compensatory postural adjustment, Postural control, Balance assessment, Side-lying hip abduction drop

## Abstract

This study was conducted to examine cortical and neuromuscular features of anticipatory and compensatory postural control during a side-lying hip abduction drop task and their relationship with standing balance. Twenty-six healthy young adults performed a side-lying drop task, single-leg standing (SLS) and a lateral step-down test (LSDT). During the side-lying drop, electroencephalography, electromyography, and pelvic accelerometry were recorded, and indices for the anticipatory postural adjustment (APA) and compensatory postural adjustment (CPA) phases were calculated. Elastic net and correlation analyses were used to examine associations with standing balance outcomes. For SLS, relatively high variable importance with non-zero standardized regression coefficients was observed for CPA-phase internal oblique/transversus abdominis activity, APA-phase pelvic acceleration Root Mean Square, CPA-phase gluteus medius activity, the C3 N1 mean potential, and CPA-phase multifidus activity. In contrast, model performance for LSDT was poor, and no variable was considered a useful predictor. Correlations of corresponding postural control variables across tasks were not significant. These findings suggest that a side-lying drop task captures task-specific cortical and neuromuscular features related to static standing balance, particularly compensatory trunk and hip muscle activity, pelvic acceleration responses, and cortical processing reflected by the N1 component. This recumbent task may therefore serve as a simple probe of sensorimotor control relevant to balance, although its applicability to dynamic balance remains limited.

## Highlights

• Side-lying drop task reflects anticipatory and compensatory postural control.
• Single-leg standing balance was associated with pelvic acceleration, CPA muscle activity, and C3 N1.
• C3 N1 was more informative than the readiness potential in this task.
• No meaningful prediction was observed for the lateral step-down test.
• A recumbent drop task may reflect neural and muscular control relevant to balance.

## Graphical Abstract

**Figure:**
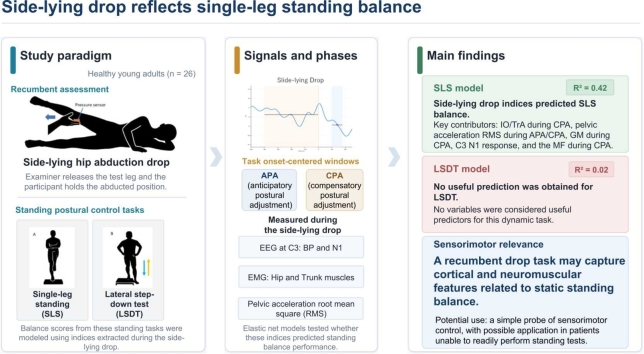

## 1 Introduction

Appropriate postural control is essential for stabilizing motor control and performance during functional movements, such as standing and walking, as well as during applied movements such as sports activities. A key postural control mechanism is anticipatory postural adjustment (APA). During movement initiation, APA operates proactively to counteract the postural perturbations expected to occur as a consequence of the intended movement (Bouisset and Do, 2008). Impairments in APA reportedly cause postural instability during voluntary movements (Horak, 2006, Kubicki et al., 2012), suggesting that APA quality influences motor control stability during movement. Conversely, compensatory postural adjustment (CPA) refers to reactive postural responses that occur to restore or maintain body stability. Both APA and CPA rely on the reciprocal regulation of sensory information and motor output to achieve postural control. Previous studies have shown that during rapid unilateral arm elevation, the muscle activity of the biceps femoris, a postural muscle, precedes that of the deltoid, the prime mover, as an APA to compensate for body sway (Bouisset and Do, 2008). Furthermore, during the transition from double-leg standing to single-leg standing (SLS), a greater center-of-pressure (COP) displacement toward the non-supporting leg side during the APA phase and a larger and faster COP displacement toward the supporting leg side during the transition phase as a CPA have been reported (Sabashi et al., 2021). Collectively, these findings demonstrate that APA and CPA occur during standing and are associated with postural stability.

Recently, APA has also been investigated in body positions other than standing. Anticipatory postural responses during lower-limb movements in the supine position are associated with postural control during standing (Lima-Pardini et al., 2017). Specifically, anticipatory ground reaction forces generated by the contralateral lower limb during lower-limb raising in the supine position are significantly correlated with ground reaction forces generated during gait initiation in standing. Moreover, lower-limb raising in the supine position reportedly elicits activation in brain regions involved in motor planning and execution associated with anticipatory postural control (Smith et al., 2023). Focusing on the relationship between functional assessments performed in the supine position and dynamic postural control performed during standing, studies have reported that proprioceptive assessment in the supine position is associated with dynamic postural control stability during standing (Bellisle et al., 2024). Further, an increased number of repeated lower-limb extension raises performed in the supine position has been associated with gait independence (Yamauchi et al., 2018). Altogether, these findings suggest that lower-limb movements in the supine position are related to both APA and dynamic postural control and may serve as an assessment reflecting postural control during standing.

As a CPA-related assessment, limb drop tests have attracted attention. A previous study reported the Reactive Leg Drop Test, which evaluates sensorimotor responses during dropping and subsequent holding of the lower limb from a seated knee-extension position, as a novel assessment method (Soucie, 2022. This test assesses the sensorimotor integration required for postural stability during standing, and its association with standing balance has been reported). A similar movement pattern is the Dunatelli Drop Test, in which the limb is dropped and subsequently held from a hip-abducted position in a side-lying position (Gowda et al., 2014). The Dunatelli Drop Test has been used to evaluate gluteus medius (GM) muscle strength; however, its relationship with sensorimotor function has not been examined. Therefore, the relationship between a lower-limb drop test performed during Side-lying hip abduction drop (Side-lying drop) and postural control or standing postural balance remains unclear.

Side-lying hip abduction movements have been used to evaluate muscle activation patterns involving not only the hip abductor muscles but also the pelvic and trunk musculature (Kim and Kim, 2018). In particular, the muscle activation balance between the GM and the tensor fasciae latae (TFL) is important for efficient hip motor control. In addition, pelvic and trunk muscles, including the quadratus lumborum (QL), internal oblique (IO), external oblique, erector spinae, and multifidus (MF), are involved during side-lying hip abduction (Kim and Kim, 2018)and are known to influence the appearance of compensatory movements and changes in muscle activation patterns during the task. Lateral control depends on the balance between the GM and TFL, as well as the coordination of muscles responsible for pelvic and trunk stabilization. Lateral control has been evaluated using SLS (Sabashi et al., 2021) and the Lateral Step-Down Test (LSDT) (Kim et al., 2016) and is also important for postural and motor control from the perspective of dynamic stability during activities, such as gait. Side-lying hip abduction offers the advantage of allowing evaluation of compensatory movements and muscle activation balance, while allowing assessment of muscle groups related to functional movements (i.e., SLS and gait) under relatively low postural-load demands.

Recent attention has focused on the possibility that postural control occurring during lower-limb movements in recumbent positions, as well as during non-standing assessments such as the Reactive Leg Drop (RLD) test performed in sitting, may reflect APA and CPA occurring during standing and postural control function. However, whether the Side-lying drop reflects postural control function during standing remains unclear. The Side-lying drop is a movement likely associated with lateral postural control. In addition, because the exercise is performed in a side-lying position, it imposes lower postural stability demands than standing tasks. Therefore, similar to the RLD test, it may be applicable in clinical practice as a simple method for evaluating postural balance ability. Accordingly, this study aimed to characterize cortical and neuromuscular features of anticipatory and compensatory postural control during a side-lying hip abduction drop task and to clarify their relationship with postural stability during standing. Based on previous findings showing that APA assessment in the anteroposterior direction in the supine position is associated with APA during gait initiation in standing (Smith et al., 2023), we hypothesized that participants exhibiting appropriate electroencephalographic (EEG) preparatory activity (readiness potential) during the APA phase of the Side-lying drop, along with appropriate muscle activation patterns and pelvic stabilization, would demonstrate less body sway and better postural stability during SLS and the LSDT. We further hypothesized that postural control indices during the APA and CPA phases of the Side-lying drop would be associated with postural control indices during the APA and CPA phases of SLS and the LSDT.

## 2 Materials and methods

### 2.1 Participants

The participants were 30 healthy young adults (age, 20.0 ± 0.6 years; 20 men and 10 women). To minimize the influence of laterality associated with foot dominance on the measurement data, only participants with right-foot dominance were included. Exclusion criteria included the presence of pain affecting postural control task performance and a history of orthopedic or central nervous system disorders. This study was approved by the Ethics Committee of Kyoto Tachibana University (Approval No. 25–64). All participants received an verbal explanation of the study and provided informed consent.

### 2.2 Study Protocol

**Fig. 1:**
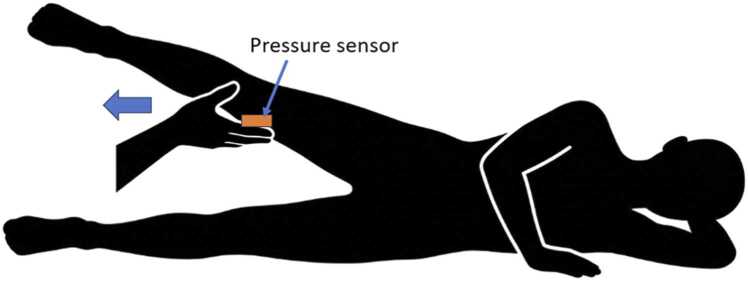
Side-Lying Hip Abduction Drop Task (Side-Lying Drop). The pressure sensor was positioned at the distal thigh to detect the timing of contact release, which was used to define task onset.

**Fig. 2:**
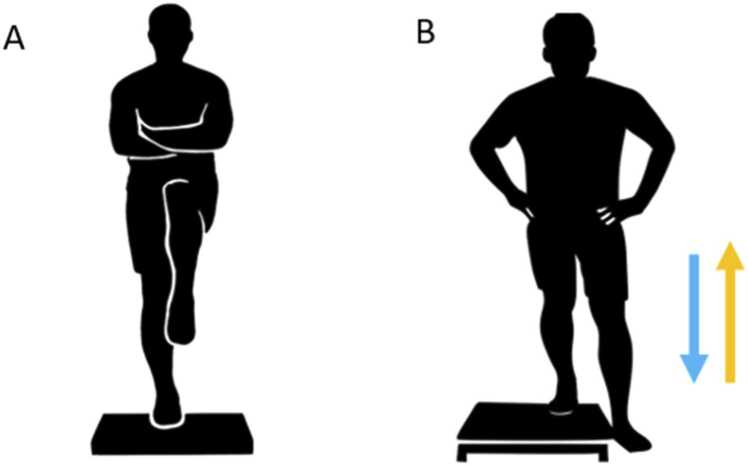
Posture Control Task. A. Single-leg stance (SLS) task. B. Lateral Step-down test (LSDT).

All participants performed the Side-lying drop task (Fig. 1), SLS, and LSDT (Fig. 2). During the Side-lying drop, EEG, electromyography (EMG), and accelerometry were simultaneously measured. During the SLS and LSDT, EEG, EMG, accelerometry, and postural sway were simultaneously measured. All devices were synchronized during data acquisition. The pressure sensor was positioned at the distal thigh to detect the timing of contact release, which was used to define task onset.

### 2.3 Side-lying hip abduction drop task (Side-Lying Drop)

For the Side-lying drop task, participants assumed a side-lying position with the nondominant leg on the lower side. The lower-side limb was positioned with the hip flexed to 45°, knee flexed to 90°, and arms crossed in front of the chest. The test-side lower limb was positioned at a starting angle of 30° hip abduction and 5° hip extension. A pressure sensor (BioSignalPlux, Plux, Inc., Lisbon, Portugal) was attached along the superior border of the medial femoral epicondyle on the test side, and the moment when the pressure on the sensor was released was defined as the movement onset for analysis. During the Side-lying drop procedure, the examiner passively positioned the participant in the starting posture. During this setup, the examiner supported the lower limb by holding it between the medial and lateral sides of the thigh to sandwich the pressure sensor placed on the medial thigh. Participants were instructed in advance to maintain the abducted position after the examiner released the supporting hand (Fig. 1). Approximately 3 s after supporting the lower limb, the examiner released the hand in response to a verbal cue. The holding time in the abducted position was set at 3 s. Three test trials were conducted after the two practice trials.

### 2.4 Single-leg standing task

To measure postural control indices during static motor control, an SLS was performed. At the starting position, participants stood at the center of the stabilometer in a double-leg stance with both feet placed at hip width, and both arms crossed in front of the chest. For the SLS, following a verbal cue, participants lifted the non-dominant-side lower limb off the floor as quickly as possible by flexing the limb, transitioning from double-leg to SLS, and maintaining this posture for 10 s (Fig. 2). To standardize attentional focus during task performance, participants were instructed to keep their posture as stable as possible while maintaining SLS. A minimum of three practice trials and three test trials were conducted, with a 3-s rest interval between trials. SLS stability was evaluated from video recordings obtained 3 m in front of the participant using a 3-point balance score based on a previous study (Kaukinen et al., 2017). A score of 0 indicated correct performance with stable posture and appropriate hip, knee, and foot alignment; 1 indicated inaccurate performance with instability and/or compensatory movement; and 2 indicated failed performance, defined as inability to perform the task or maintain one-leg standing for at least 5 s. The same examiner reviewed all recordings, and the mean score across the three trials was used for analysis.

### 2.5 LSDT

The LSDT was performed to measure the postural control indices during dynamic motor control. A stabilometer was placed on a 10 cm-high platform, and the total height of the platform, including the stabilometer, was set at 15 cm. Participants stood with both feet on the stabilometer, aligned to the edge on the side of the non-dominant foot, and placed both hands on the iliac crests. After the cue, participants stood on the dominant leg and flexed the knee of the dominant side until the heel of the non-dominant foot touched the floor; immediately after contact, the heel was lifted off the floor and returned to the starting position (Fig. 2). To standardize attentional focus during task performance, participants were instructed to maintain their posture as stable as possible throughout the LSDT movement. Five practice trials were performed, followed by five consecutive trials. LSDT performance was evaluated from video recordings obtained 3 m in front of the participant using a scoring system based on a previous study (Rabin et al., 2014). One point was assigned for each of the following movement faults: the hands leaving the iliac crests, trunk lean to either side, pelvic tilt indicating loss of a level pelvic plane, the tibial tuberosity positioned medial to the second toe, and loss of postural stability, such as contact of the non-test foot with the floor or excessive body sway. Two points were assigned when the tibial tuberosity moved medial to the medial border of the foot. The total score ranged from 0 to 6 points, representing a 7-point scale. According to Rabin et al. (2014), total scores of 0–1 indicated good movement quality, scores of 2–3 were classified as moderate movement quality, and scores of 4 or more were classified as poor movement quality. The same examiner reviewed all recordings, and the mean score across the five trials was used as a continuous outcome variable for analysis.

### 2.6 Data acquisition and analysis

To evaluate the characteristics of motor control variables during motor control tasks, EEG, EMG, and accelerometry were performed during the Side-lying drop task, and postural sway was additionally measured during the SLS and LSDT. All devices were synchronized during data acquisition, and the sampling frequency was set to 1000 Hz. Data were analyzed using MATLAB (R2025b, MathWorks, Natick, MA, USA).

**Fig. 3:**
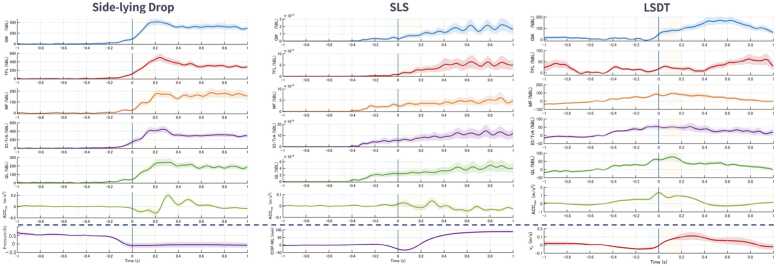
Time-series plots of postural control indices during Side-lying drop, SLS, and LSDT. Solid lines represent the group mean across participants. The anticipatory postural adjustment (APA) phase is highlighted in light blue, the compensatory postural adjustment (CPA) phase in light orange, and the baseline interval in gray. The bottom panels show the reference signals used to define movement onset (Pressure for Side-lying drop, COP-ML for SLS and Vy for LSDT). Abbreviations: GM, gluteus medius; TFL, tensor fasciae latae; MF, multifidus; IO/TrA; internal oblique/transversus abdominis; QL, quadratus lumborum: ACCres, resultant acceleration; COP-ML, mediolateral center of pressure. Vy, velocity y axis.

The movement onset and phase classification criteria for each task used in this study are described below: Each postural control index was classified by movement into APA and CPA phases. The time windows for the APA and CPA phases were defined with the movement onset criterion for each task set as T = 0 s, with the APA phase defined as −100 ms to + 50 ms and the CPA phase as + 50 ms to + 200 ms (Zhang et al., 2023). For the Side-lying drop task, the time at which the pressure sensor reached 0 N was defined as the movement onset. In the SLS, the peak COP displacement toward the non-supporting leg in the mediolateral direction, measured using a stabilometer, was visually identified as the end of the APA phase (T + 50 ms) (Sabashi et al., 2021) (Fig. 3). In the LSDT, the moment at which the acceleration measured by the accelerometer began to descend in the vertical direction was defined as movement onset (Kim et al., 2016) (Fig. 3).

#### 2.6.1 EMG

Muscle activity was measured using EMG sensors (BioSignalPlux; Plux Inc., Lisbon, Portugal). The band-pass filter was set to 10–400 Hz. The target muscles were those on the dominant side of the lower limb and trunk, and electrodes were placed over the GM, TFL, lumbar multifidus (MF), IO/transversus abdominis (TrA), and QL, which are relevant to motor control tasks. The electrode sites were as follows: GM, at the midpoint between the iliac crest and greater trochanter (Park et al., 2010); TFL, slightly lateral and 2 cm inferior to the anterior superior iliac spine (ASIS) (Lee et al., 2014); MF, 2 cm lateral to the L5 spinous process (Park et al., 2010); IO/TrA, 2 cm inferomedial to the ASIS (Park et al., 2010); and QL, 4 cm lateral to the vertebral prominence and at the midpoint between the 12th rib and iliac crest (Park et al., 2010). A reference electrode was attached to the spinal spinous process. Before electrode placement, the skin was cleaned with an alcohol swab.

A fourth-order band-pass filter (10–400 Hz) was applied to the EMG signals. The signals were full-wave rectified, and, in accordance with a previous study, the time-integrated EMG (iEMG) for each movement phase was calculated as the muscle activity index relative to T = 0: baseline (BL; (−0.50 to −0.45 s), APA (−0.10 to +0.05 s), and CPA (+0.05 to +0.20 s) phases (Zhang et al., 2023).

ΔiEMG_APA_ = (iEMG_APA_ − 3 · iEMG_BL_) / (3 · iEMG_BL_)

ΔiEMG_CPA_ = (iEMG_CPA_ − 3 · iEMG_BL_) / (3 · iEMG_BL_)

For each motor control task, the mean iEMG values across all trials were used as muscle activity indices for the statistical analyses.

#### 2.6.2 Accelerometry

An accelerometer (BioSignalPlux, Plux, Inc., Lisbon, Portugal) was used to measure the pelvic sway as a postural control index. The accelerometer was attached at the midpoint between the bilateral posterior superior iliac spines to record pelvic motion. To remove high-frequency noise, the signals were processed using a fourth-order zero-lag Butterworth low-pass filter with a cutoff frequency of 8 Hz (Kim et al., 2016). Pelvic sway was quantified by calculating the resultant acceleration from the triaxial accelerations ax(t), ay(t), and az(t) (Eq. [1]), and the root mean square (RMS) of the resultant acceleration within the APA and CPA analysis windows was calculated using the RMS_APA_ (Eq. [2]) and RMS_CPA_(Eq. [3]):

(1) a_res_(t) = √(a_x_(t)^2^ + a_y_(t)^2^ + a_z_(t)^2^)

(2) RMS_APA_ = √((1/N_APA_) Σ_t∈APA_ (a_x_(t)^2^ + a_y_(t)^2^ + a_z_(t)^2^))

(3) RMS_CPA_ = √((1/N_CPA_) Σ_t∈CPA_ (a_x_(t)^2^ + a_y_(t)^2^ + a_z_(t)^2^))

For each motor control task, the mean values of RMS_APA_ and RMS_CPA_ across all trials were used as pelvic sway indices in the statistical analyses.

#### 2.6.3 Postural Sway

To define the movement phases in the SLS, a force platform (BioSignalPlux, Plux, Inc., Lisbon, Portugal) was used to measure the COP. The COP signal was processed using a fourth-order Butterworth low-pass filter with a cutoff frequency of 250 Hz (Kim et al., 2016).

#### 2.6.4 EEG

An 8-channel dry-electrode EEG system (Altaire, Creact Inc., Japan) was used for EEG recording. EEG recording sites were configured at eight channels based on the international 10–20 system: Fp1, Fp2, Fz, C3, C4, Pz, O1, and O2. The present montage did not include Cz, despite previous EEG studies demonstrating frontocentral cortical activity involvement in the preparation and execution of lower-limb movements accompanied by APA (Varghese et al., 2016). In the present study, C3 was selected as the available left central sensorimotor derivation to evaluate cortical activity associated with the right-sided lower-limb task exploratorily. This electrode selection was based on the lateralized nature of the task and the available EEG montage, and was not intended to specifically localize lower-limb motor cortical activity. The reference was the bilateral earlobe average obtained from A1 and A2 ear clips. Electrode attachment sites were cleaned before EEG-device placement. Measurements were conducted in a quiet environment with participants’ eyes open while wearing earplugs and earmuffs to minimize auditory stimulation.

**Fig. 4:**
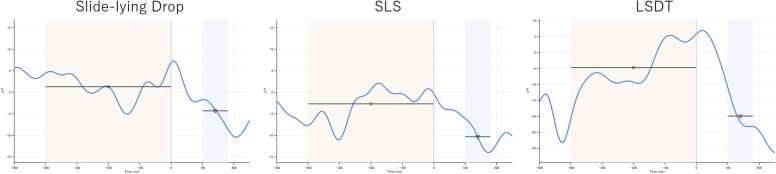
Representative event-related potential waveforms illustrating the BP and N1 components during the Side-lying drop, SLS, and LSDT. Time is shown relative to task onset (0 ms, vertical line). The beige shaded region indicates the predefined BP time window (−400–0 ms), and the light blue shaded region indicates the predefined N1 time window (+100 to +180 ms). Horizontal lines represent the mean potentials used for BP and N1 quantification within each time window. Amplitude is expressed in μV.

For EEG preprocessing, a 1–30 Hz band-pass filter was applied, followed by independent component analysis. Artifact components, including ocular and EMG-related artifacts, were removed with reference to automatic classification using ICLabel (Pion-Tonachini et al., 2019). For EEG analysis indices, the Bereitschaftspotential (BP) and N1 component were used as the event-related potential indices for the APA and CPA phases, respectively. BP was treated as a slow negative potential associated with motor preparation and was calculated by subtracting the BL mean potential (−1500 to −1300 ms) from the mean potential during the preparatory period (−400–0 ms) (Braquet et al., 2020). The N1 component was quantified as the mean potential in the 100–180 ms window relative to the pre-onset baseline from −1000 to −500 ms (Mierau et al., 2015). The 100–180 ms window was selected based on previous studies showing that the balance N1 typically occurs approximately 100–200 ms after perturbation onset (Payne and Ting, 2020). An immediate pre-onset interval was not used as the N1 baseline because it may include premovement negative shift (Fig. 4). Time is shown relative to task onset (0 ms, vertical line). The beige shaded region indicates the predefined BP time window (−400–0 ms), and the light blue shaded region indicates the predefined N1 time window (+100 to +180 ms). Horizontal lines represent the mean potentials used for BP and N1 quantification within each time window. Amplitude is expressed in μV.

### 2.7 Statistical analyses

To examine the relationships between the balance scores obtained in each standing postural control task and the indices measured during the Side-lying drop task, elastic net analyses were performed with the balance score of each motor control task as the dependent variable and the measurement indices obtained during the Side-lying drop task as explanatory variables. For each elastic net model, the variable importance, standardized regression coefficients, and model fit indices were calculated, and the models for the motor control tasks were compared. The elastic net is a regularized linear regression method that uses a mixed penalty of L1 (LASSO) and L2 (ridge). Shrinking the coefficients while setting some coefficients to zero enables variable selection and is suitable for stable modeling, even when highly correlated explanatory variables are present (Zou and Hastie, 2005). In addition, to examine the relationships of corresponding postural control indices between the Side-lying drop and SLS/LSDT in the APA and CPA phases, correlation analyses were conducted for postural control indices across tasks. The significance level was set at 5%. Statistical analyses were performed using the JASP software (ver. 0.19.2.0).

Data quality was evaluated following offline preprocessing. Although gross signal acquisition was confirmed during online monitoring, datasets were excluded when the predefined APA/CPA indices could not be reliably computed due to residual EEG artifacts after ICA, unstable baseline activity, excessive amplitude fluctuations, EMG artifacts despite filtering and rectification, or an insufficient number of usable trials. Usable trials were defined as those in which task onset was clearly identifiable and both predefined EEG and EMG signals satisfied the predefined quality criteria for analysis. Participants were excluded when reliable indices could not be obtained after offline quality control. Although 30 participants were initially enrolled in this study, four demonstrated poor measurement quality and were excluded. Consequently, 26 participants (18 men and eight women) were included in the analyses.

## 3 Results

### 3.1 Participant characteristics

Table 1 summarizes the participant characteristics. The median balance scores were 1.0 and 2.5 for the SLS and LSDT, respectively.

**Table 1:** Participant characteristics.

Variables	Median (interquartile range)
Age (years)	20.0 (1.0)
Height (cm)	167.0 (11.0)
Body weight (kg)	57.0 (11.5)
BMI (kg/m²)	20.8 (1.9)
SLS balance score (points)	1.0 (1.8)
LSDT balance score (points)	2.5(4.0)

BMI, body mass index; SLS, Single-Leg Standing; LSDT, Lateral Step-Down Test.

### 3.2 Elastic net analysis using motor control variables in the side-lying drop task for the SLS balance score

Elastic net analysis using the motor control variables from the Side-lying drop task to predict the balance score of the SLS showed the following model performance: mean squared error (MSE) = 2.04, root MSE (RMSE) = 1.43, and R^2^ = 0.42. Regarding the variable importance for the SLS balance score (Table 2), the highest values were observed, in descending order, for CPA-phase IO/TrA activity, APA-phase pelvic acceleration RMS, CPA-phase GM activity, CPA-phase pelvic acceleration RMS, the C3 N1 mean potential, and CPA-phase MF activity. Variables with non-zero standardized regression coefficients were therefore interpreted as selected predictors, whereas those with coefficients equal to zero were considered non-selected.

**Table 2:** Elastic net analysis using motor control variables in the Side-lying drop task for the balance score of the SLS.

Side-lying Drop variables	Variable importance	Standardized regression coefficient (β)
CPA phase: IO/TrA	1.065	0.223
APA phase: Pelvic acceleration RMS	1.052	−0.192
CPA phase: GM	1.051	−0.197
CPA phase: Pelvic acceleration RMS	0.985	−0.020
C3 N1 mean potential	0.984	0.016
CPA phase:MF	0.980	−0.197
APA phase: IO/TrA	0.980	0
CPA phase: TFL	0.980	0
APA phase: TFL	0.980	0
APA phase: MF	0.980	0
APA phase:QL	0.980	0
APA phase: GM	0.980	0
CPA phase: QL	0.980	0
C3 BP mean potential	0.980	0

*Variables are listed in descending order of importance.

APA, anticipatory postural adjustments; CPA, compensatory postural adjustments; GM, gluteus medius; TFL, tensor fasciae latae; IO/TrA. internal oblique/transversus abdominis; MF, multifidus; QL, quadratus lumborum; RMS, root mean square; BP, Bereitschaftspotential.

### 3.3 Elastic net analysis using motor control variables in the side-lying drop task for the LSDT balance score

Elastic net analysis using the motor control variables from the Drop test to predict the LSDT balance score yielded the following model performance: MSE = 2.68, RMSE = 1.64, and R^2^ = 0.02. The variable importance values for the LSDT balance score are shown in Table 3; however, because model performance was extremely low, no variables were considered useful predictors.

**Table 3:** Elastic net analysis using motor control variables in the Side-lying drop task for the LSDT balance score.

Side-lying drop variables	Variable importance	Standardized regression coefficient (β)
APA phase: Pelvic acceleration RMS	2.76	−0.57
C3 BP mean potential	2.70	−0.00
CPA phase: GM	2.62	−0.17
APA phase: GM	2.50	0
APA phase: TFL	2.50	0
APA phase: QL	2.50	0
APA phase: MF	2.50	0
APA phase: IO/TrA	2.50	0
CPA phase: Pelvic acceleration RMS	2.50	0
CPA phase: TFL	2.50	0
CPA phase: IO/TrA	2.50	0
CPA phase: MF	2.50	0
CPA phase: QL	2.50	0
C3 N1 mean potential	2.50	0

APA, anticipatory postural adjustments; CPA, compensatory postural adjustments; GM, gluteus medius; TFL, tensor fasciae latae; IO/TrA. internal oblique/transversus abdominis; MF, multifidus; QL, quadratus lumborum; RMS, root mean square; BP, Bereitschaftspotential.

*Variables are listed in descending order of importance.

### 3.4 Correlation analysis of corresponding postural control variables across tasks in the side-lying drop, SLS, and LSDT

The results of the correlation analysis of the postural control variables across the Side-lying drop and SLS showed no significant correlations for any variable across tasks (Table 4). Similarly, in the correlation analysis of postural control variables across the Side-lying drop and the LSDT, no significant correlations were observed for any variable across tasks (Table 4).

**Table 4:** Correlation analysis of corresponding postual control variables between the Side-lying drop task and each postural control task.

Side-lying drop		SLS		LSDT	
		Correlation coefficient	P-value	Correlation coefficient	P-value
Phase	Postural control variables				
APA phase	C3 BP mean potential	0.17	0.42	0.30	0.13
	Pelvic acceleration RMS	0.15	0.46	0.29	0.14
	GM	0.15	0.46	0.12	0.55
	TFL	0.07	0.72	0.31	0.13
	IO/TrA	−0.05	0.81	0.19	0.36
	MF	−0.05	0.83	0.19	0.36
	QL	0.08	0.70	−0.13	0.51
CPA phase	C3 N1 mean potential	0.38	0.06	−0.06	0.79
	Pelvic acceleration RMS	0.16	0.44	0.18	0.38
	GM	−0.11	0.60	0.04	0.86
	TFL	−0.12	0.57	0.06	0.77
	IO/TrA	0.006	0.98	0.27	0.19
	MF	−0.05	0.81	−0.10	0.63
	QL	0.11	0.61	−0.30	0.14

SLS, Single-Leg Standing task; LSDT, Lateral Step-Down Test; APA, anticipatory postural adjustments; CPA, compensatory postural adjustments; GM, gluteus medius; TFL, tensor fasciae latae; IO/TrA. internal oblique/transversus abdominis; MF, multifidus; QL, quadratus lumborum; RMS, root mean square; BP, Bereitschaftspotential.

## 4 Discussion

This study examined whether a side-lying hip abduction drop task captures cortical and neuromuscular features of postural control that are relevant to standing balance. These findings suggest that postural control indices obtained during the Side-lying drop task are related to the balance score in the SLS. In particular, the elastic net analysis identified CPA-phase IO/TrA activity, APA-phase pelvic acceleration RMS, CPA-phase GM activity, the C3 N1 mean potential, and CPA-phase MF activity as variables with relatively high significance and non-zero coefficients. These findings suggest that compensatory trunk and hip muscle activity, pelvic acceleration responses, and cortical processing during the CPA phase may underlie the association between the Side-lying drop task and static standing balance performance. Conversely, for LSDT balance, model performance was poor, indicating that postural control indices from the Side-lying drop task were not predictive of performance. Moreover, analyses of individual postural control indices revealed no significant correlations between the Side-lying drop task and the static/dynamic standing postural control tasks. Collectively, these findings suggest that the Side-lying drop task may be applicable for evaluating SLS balance, although the direct relationships between postural control indices across tasks appear weak.

In this study, postural control indices obtained during the Side-lying drop task could predict SLS balance scores; however, correlation analyses showed no associations among the motor control variables across tasks. Based on the standardized regression coefficients for EEG, the N1 component, which reflects EEG activity during the CPA phase, was extracted as an important predictor. The positive standardized regression coefficient indicates that a smaller negative amplitude of the N1 component (i.e., a less pronounced cortical response)was associated with poorer balance scores, whereas the BP component, which reflects EEG activity during the APA phase, was not selected. In the postural control domain targeted in this study, the N1 component, often referred to as the perturbation-evoked N1, reportedly responds to unexpected postural perturbations (Näätänen and Picton, 1987). Although localization to the supplementary motor area has previously been demonstrated as an N1-related neural substrate, synchronized activity across a broader network, including the anterior cingulate, sensorimotor, and parietal cortices, has also been suggested, and N1 activity has been interpreted as an expression of a motor association/monitoring/updating system, with the supplementary motor area as a hub (Braquet et al., 2020). In addition, the N1 component is reportedly influenced by factors such as perceived threat and attention to postural sway, reflecting increased cortical involvement in response to greater postural error or compensatory demand (Braquet et al., 2020). Therefore, N1 likely functions in postural control as a cortical monitoring and updating process for postural errors, and postural control tasks requiring error correction in response to external perturbations, such as the drop test, may more likely reflect the N1 component. Regarding BP characteristics, the BP is typically observed during self-initiated voluntary movements, but is diminished when the same movement is externally cued (McGill et al., 2003). Taken together, because the Side-lying drop task used in this study was dominated by reactive motor components dependent on gravity and external perturbation during postural maintenance, we speculate that responses were more strongly observed in the N1 component, which is closely related to postural sway, whereas BP responses, more closely linked to self-initiated movement, were attenuated.

Regarding the relationship between the Side-lying drop task and the SLS balance score, several variables with high importance were identified in relation to muscle activity. CPA-phase IO/TrA activity demonstrated the highest variable importance, and the positive standardized regression coefficient indicated that greater IO/TrA activity during the CPA phase was associated with poorer SLS balance scores. Conversely, CPA-phase GM and MF activity exhibited negative standardized regression coefficients, indicating that reduced GM and MF activation during the CPA phase was associated with poor SLS balance scores. Moreover, reduced pelvic acceleration RMS, particularly during the APA phase, was associated with poorer SLS balance scores. Overall, these findings suggest that poor SLS balance may be characterized by increased compensatory abdominal muscle activity, reduced local hip and lumbar muscle activity, and diminished pelvic acceleration responses during the Side-lying drop task. In postural control, coordinated activity of the multifidus and pelvic muscles is required for local stability of the spine and pelvis. Lumbar and pelvic stability are influenced by the interaction between local trunk muscles, including the MF and TrA, as well as supraspinal motor control and proprioceptive processes (Meier et al., 2019). The brain modulates muscle activation patterns based on an optimal control model, relying on internal models that integrate sensory feedback (Meier et al., 2019). In addition, increased co-contraction or excessive stiffening in response to postural perturbation has been associated with unsuccessful postural control (Falk et al., 2022). Therefore, participants with insufficient fine-tuning of CPA-phase GM and MF activity during the Side-lying drop task may have compensated through excessive IO/TrA activity, reflecting a strong “tight control” strategy and resulting in poor SLS balance. Furthermore, reduced pelvic acceleration RMS during the APA phase was associated with poorer SLS balance scores. APA is a predictive postural control mechanism that prepares the body for efficient execution of subsequent CPA (Santos et al., 2010). APA scaling is also modulated by postural threat and task demands (Phanthanourak et al., 2023), and age-related differences in APA have also been reported (Duarte et al., 2022). Therefore, reduced pelvic acceleration responses during the APA phase may reflect insufficient preparatory pelvic motion for the subsequent compensatory response. Participants demonstrating a tightly regulated control strategy during the APA phase may exhibit reduced flexibility and diminished capacity for fine-tuned muscle modulation during the CPA phase, which in turn may contribute to poorer SLS balance performance. Taken together, these findings suggest that, in static postural control tasks such as SLS, appropriate pelvic motor responses during the APA phase and optimized compensatory muscle activity during the CPA phase in the Side-lying drop task may be critical for balance control.

We examined a predictive model for the relationship between the Side-lying drop task and LSDT; however, the model showed poor performance, suggesting that although the Side-lying drop task could predict the balance score of the SLS to a limited extent, it is not suitable for predicting the LSDT balance score. We consider the differences in task characteristics across tasks to be a major contributor to this result. Regarding postural balance, improvements in balance ability are reportedly specific to practiced movements and show little transfer to novel tasks involving different postures or stimuli (Meier et al., 2019), indicating that the relationship in postural control between tasks is likely stronger when the tasks involve similar postures and motor control demands. The Side-lying drop task is a static postural control task requiring maintenance of the hip in an abducted position, and the SLS is likewise a static postural control task requiring maintenance of an SLS posture. In contrast, the LSDT is a dynamic postural control task, as it requires postural control while lowering the lower limb from a step. In addition, previous studies have reported no significant correlation between the test results for static and dynamic stabilities (Kümmel et al., 2016). From a functional perspective, the SLS primarily requires lateral pelvic stability (Karimi, Solomonidis, 2011), and the side-lying hip abduction movement similarly requires lateral stability mediated by GM activity (Kiyota and Fujiwara, 2014), indicating a shared functional characteristic. In contrast, the LSDT requires both lateral and anterior-posterior stability because it involves flexion-extension movements of the lower limbs and trunk (Cynn et al., 2006). Collectively, compared with the SLS, the LSDT has less functional similarity to the Side-lying drop task and differs in the required direction of pelvic postural control. Therefore, the Side-lying drop task may not be appropriate for predicting the LSDT balance score.

Recently, postural control assessment in the side-lying position has been suggested as a potential approach for predicting standing postural control ability. In the present study, although only to a limited extent, the Side-lying drop task was able to predict the balance score of the SLS; however, no correlations were observed with individual postural control indices. In addition, no association was observed between the Side-lying drop task and the LSDT. These findings indicate that, although postural control assessment in a recumbent position was not associated with postural control indices measured during standing, it may still be useful for predicting SLS balance performance. Previous evidence also indicates associations between recumbent-position assessments using the straight-leg raise, standing posture, and gait performance (Smith et al., 2023). Accordingly, the Side-lying drop task used in this study may have potential clinical utility as an assessment tool for predicting not only SLS balance, but also standing posture and movement performance, even in patients who find it difficult to get out of bed.

This study has some methodological limitations. First, participants were limited to healthy young adults. Aging and the presence of pain reportedly affect central nervous system activity, muscle activity, and kinematic indices (Ahmad and Barkana, 2025, Bosse et al., 2012, Hsiao and Cho, 2012); therefore, further verification is required before generalizing these findings to older adults or individuals with pain. Second, surface EMG sensors were used for EMG measurements. Although the approach was consistent with previous studies, some target muscles were located relatively deep, and the influence of crosstalk from adjacent muscles cannot be ruled out. Third, balance scores for each task were based on the raters’ subjective and visual evaluations of video recordings, which inherently depend on subjective judgment. Therefore, subjective bias may have influenced the balance scores. Fourth, the EEG montage employed in this study did not include Cz, which is considered generally more appropriate for assessing lower-limb sensorimotor cortical activity. Therefore, the C3 activity analyzed in this study should be interpreted as an exploratory central sensorimotor index rather than as a localized measure of lower-limb motor cortical activity. Future studies should use a higher-density EEG montage, including Cz, to precisely examine lower-limb-related cortical activity. Fifth, in the LSDT analysis, movement onset was defined as the onset of pelvic descent in accordance with previous studies. However, because participants were already in a SLS position at that point, deriving postural control indices that strictly reflected the APA phase may have been difficult. Future studies should refine the analytical methods, including phase segmentation and measurement sites, and examine a broader range of participants, including different age groups and individuals with different clinical conditions, to facilitate clinical application.

These findings suggest that a Side-lying drop task captures task-specific cortical and neuromuscular features related to static standing balance. In particular, compensatory IO/TrA, GM, and MF activity, APA-phase pelvic acceleration responses, and the C3 N1 component were associated with SLS balance, whereas analogous predictive relationships for the LSDT were limited. These findings support the use of this recumbent task as a probe of sensorimotor control relevant to static balance, while further studies are needed to determine its clinical applicability and generalizability.

## Ethical approval statement

This study was approved by the Ethics Committee of Kyoto Tachibana University (Approval No. 25–64). All participants received an oral explanation of the study and provided informed consent.

## Funding

This work was supported by a grant from JSPS KAKENHI (grant number 23K165580 and 25K141790).

## CRediT authorship contribution statement

**Takayuki Kodama:** Writing – review & editing, Supervision. **Hayato Shigetoh:** Writing – review & editing, Visualization, Validation, Supervision, Software, Resources, Project administration, Methodology, Investigation, Funding acquisition, Formal analysis, Data curation, Conceptualization. **Hayato Wakabayashi:** Writing – review & editing, Investigation. **Junya Miyazaki:** Writing – review & editing, Supervision, Resources, Project administration, Funding acquisition. **Utaha Aoki:** Writing – review & editing, Investigation. **Kota Maeda:** Writing – review & editing, Investigation. **Kota Kitamura:** Writing – original draft, Visualization, Validation, Methodology, Investigation, Formal analysis, Data curation, Conceptualization. **Naoya Shimoda:** Writing – original draft, Visualization, Validation, Methodology, Investigation, Formal analysis, Data curation, Conceptualization.

## Declaration of Competing Interest

The authors declare that they have no conflict of interest.

## Data Availability

The original contributions presented in this study are included in the article. Further inquiries can be directed to the corresponding authors.
